# Editorial: Towards an Understanding of the Relationship Between Spatial Processing Ability and Numerical and Mathematical Cognition

**DOI:** 10.3389/fpsyg.2020.00014

**Published:** 2020-01-29

**Authors:** Firat Soylu, Sharlene D. Newman

**Affiliations:** ^1^Educational Psychology Program, The University of Alabama, Tuscaloosa, AL, United States; ^2^The Department of Psychology, The University of Alabama, Tuscaloosa, AL, United States

**Keywords:** numerical cognition, mathematical development, learning, spatial ability, mental number line, snarc, mental rotation

Engaging children in spatial thinking early may be important given that research as far back as Bingham's 1937 Aptitudes and Aptitude Testing reported that spatial thinking is associated with success in science, mathematics, and engineering fields. More specifically, recent research suggest a strong relationship between spatial ability and mathematics: Studies have found that performance in spatial tasks, for example mental rotation and visuospatial working memory, are correlated with mathematics achievement in school age children, that spatial representations are crucial in mathematical learning, and that spatial ability is closely related to numerical development. As a result, there have been recommendations to promote spatial play in preschool and incorporate spatial reasoning into elementary school curricula.

The mechanisms that underlie the relationship between spatial ability and mathematics are unclear. While there is some evidence to suggest that spatial ability can be trained, there are still questions concerning how best to train spatial ability. Further, how spatial representations for mathematical concepts play a role during the learning process is still under consideration. Therefore, this Research Topic presents 10 articles focusing on mechanisms underlying use of spatial representations in numerical cognition, the relation between spatial and mathematical abilities, and effectiveness of learning designs and interventions to foster mathematical performance, mediated by improvements in spatial abilities.

An important spatial concept that is used to represent numerical magnitudes is the *Mental Number Line (MNL)*. There are five studies in this Research Topic that focus on MNL, and the associated Spatial-Numerical Association of Response Codes (SNARC) and Numerical Congruity (NCE) effects. Daker and Lyons found that symbolic number comparison and non-verbal reasoning predicted a unique variance in a number-line estimation task, compared to approximate number processing ability, in first-grade children. They also found that the relation between symbolic number comparison and number-line ability was stronger for male students then female. Based on these results they argued for promoting children's understanding of symbolic rather than non-symbolic numerical magnitudes when learning from number-lines in the classroom. Fischer et al. found that while the different response modes (whole-body movements, hand movements, and verbal responses) did not affect the NCE and the SNARC effect, the presentation of a number line affected the two effects in opposite ways. While a larger SNARC effect was observed when a number line was presented, the NCE was only observed when no number line was presented, suggesting that the two effects reflect distinct processes. The SNARC effect is usually thought to reflect the existence of a horizontal mental number line, with values increasing from left to right. Some studies also show other alternative organizations of numbers in the two-dimensional space, in particular in a vertical fashion (“more is up”). Sixtus et al. investigated number representation over both vertical and horizontal dimensions within the same task-context. They found that the horizontal aspect of the spatial-numerical association did not have an affect on the behavioral performance, while the vertical one did, with higher performance when larger numbers were presented in the upper space. They concluded that numbers are conceptually associated with the vertical dimension when they are presented in a two-dimensional space. Podwysocki et al. investigated if the mental number line is unique to numbers or instead a general phenomenon that applies to any ordered list, by comparing performance with number and letter stimuli. They found comparable spatial-order effects for numbers and letters. They suggested that the mental line representation underlie ordered lists in general and is not unique to numbers, and the mental number line is supported by a common representation for all ordered sequences. Toomarian et al. investigated the relation between SNARC effect, fraction performance, and general mathematics ability. They replicated the SNARC effect with fractions and found that the individual SNARC effects correlated with performance on a fraction number-line estimation (NLE) task. Further, even though the NLE—but not SNARC—performance predicted scores in a fractions test and basic standardized mathematics performance, it did not predict algebra scores, implying that NLE may not be recruited for higher-order mathematical concepts.

In addition to spatial-numerical representations in numerical cognition, in particular the mental number line, there are other important aspects of the relation between spatial ability and mathematical thinking, some which were the foci for three further articles in this Research Topic. Haynes et al. reported that perception of vertical, a spatial orientation ability important to physical activity, is associated with male numeracy and female writing scores in children. Visual field independence (FI)—the degree of not being influenced by the background during perception of vertical—was found to be most strongly associated with numeracy, among other academic measures (i.e., reading, writing). Based on these results, the authors argued that systems for small visual angle spatial performance might be involved in number processing. van Tetering et al. presented results from a large scale study focusing on the relation between mental rotation ability and mathematical performance with 7 to 12 year old children. They replicated the finding that mental rotation ability correlates with mathematical achievement, especially for boys. They also reported sex differences both in spatial ability and mathematics performance in the younger group (grades 2, 3, and 4) but not in the older one (grades 5 and 6). Based on these findings, the authors emphasized the importance of early experiences with spatial activities and play. Cueli et al. compared how non-symbolic and spatial magnitude comparison skills relate to mathematics ability in younger children. They reported that only the non-symbolic magnitude comparison scores predicted mathematics ability, pointing to the importance of training with non-symbolic magnitude tasks in early years of schooling.

Finally, another crucial aspect of the relation between spatial thinking and mathematical cognition is learning designs that involve spatial thinking and play, in order to promote mathematical learning and performance. Two studies in this Research Topic addressed this issue: Pires et al.'s intervention study with first-graders shows that technologically enhanced tangible manipulatives provide some advantages over explicitly virtual manipulatives. Children more readily interact with tangible manipulatives and the extent of interaction with manipulatives predict performance outcomes. Further, Volpe and Gori provide a set of principles on the use of multisensory technologies for learning and reflect on how multisensory technologies can be used in education.

## Author Contributions

All authors listed have made a substantial, direct and intellectual contribution to the work, and approved it for publication.

### Conflict of Interest

The authors declare that the research was conducted in the absence of any commercial or financial relationships that could be construed as a potential conflict of interest.

